# Determinants of the population health distribution: an illustration examining body mass index

**DOI:** 10.1093/ije/dyz245

**Published:** 2020-01-13

**Authors:** David Bann, Emla Fitzsimons, William Johnson

**Affiliations:** 1 Centre for Longitudinal Studies, University College London Institute of Education, London, UK; 2 School of Sport, Exercise and Health Sciences, Loughborough University, Loughborough, UK

**Keywords:** Epidemiological methods, distributions, quantile regression, body mass index, obesity

## Abstract

Most epidemiological studies examine how risk factors relate to average difference in outcomes (linear regression) or odds of a binary outcome (logistic regression); they do not explicitly examine whether risk factors are associated differentially across the distribution of the health outcome investigated. This paper documents a phenomenon found repeatedly in the minority of epidemiological studies which do this (via quantile regression): associations between a range of established risk factors and body mass index (BMI) are progressively stronger in the upper ends of the BMI distribution. In this paper, we document this finding and provide illustrative evidence of it in the 1958 British birth cohort study. Associations of low childhood socio-economic position, high maternal weight, low childhood general cognition and adult physical inactivity with higher BMI are larger at the upper end of the BMI distribution, on both absolute and relative scales. For example, effect estimates for socio-economic position and childhood cognition were around three times larger at the 90th compared with 10th quantile, while effect estimates for physical inactivity were increasingly larger from the 50th to 90th quantiles, yet null at lower quantiles. We provide potential explanations for these findings and discuss implications. Risk factors may have larger causal effects among those in worse health, and these effects may not be discovered when health is only examined in average terms. In such scenarios, population-based approaches to intervention may have larger benefits than anticipated when assuming equivalent benefit across the population. Further research is needed to understand why effect estimates differ across the BMI outcome distribution and to investigate whether differential effects exist for other physical and mental health outcomes.

## Introduction

**Key Messages**

- In the minority of epidemiological studies that employ quantile regression, risk factors for higher BMI appear to have stronger effects at the upper BMI levels.
- We demonstrate this phenomenon using the 1958 British birth cohort study. Associations of low childhood socio-economic position, high maternal weight, low childhood general cognition and adult physical inactivity with higher BMI are larger at the upper end of the BMI distribution.
- A number of potential explanations for such differences are discussed, as are potential implications. Where risk factors have larger effects amongst those in worse health, population-based approaches to intervention may have larger benefits than anticipated when assuming equivalent benefit across the population.
- Further research is needed to (1) understand reasons for differential magnitude of effect estimates across the BMI outcome distribution and (2) examine whether such differential effects exist for other physical and mental health outcomes.

**Figure 1.**
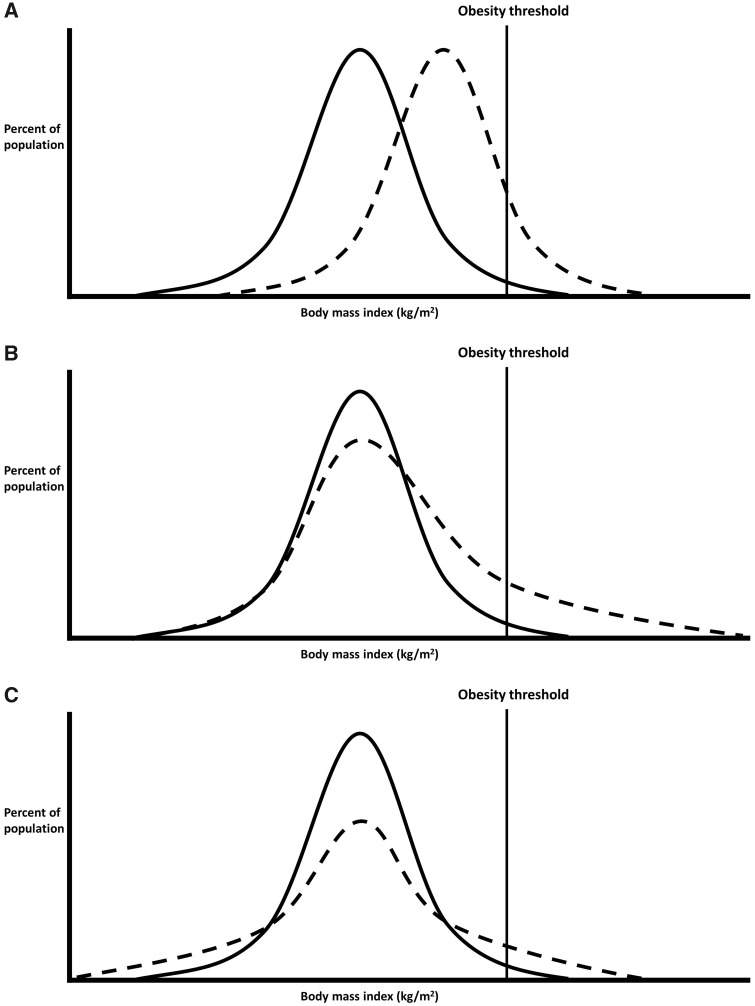
Comparisons between groups: mean differences only (A), mean differences driven particularly by differences at the upper quantiles (B), no mean difference yet different distributions (C). Note that variance is also higher in (B) and (C) than in (A). Figure adapted from Beyerlein A. Quantile regression—opportunities and challenges from a user's perspective. Am J Epidemiol 2014; 180(3): 330–31, by permission of Oxford University Press.

Epidemiology is concerned with understanding the distribution of health in a given population—first in describing it, and second in understanding its determinants.^1^^,^^2^ Yet in the majority of aetiological applications, the *distribution* of health is seldom of explicit focus regardless of the analytical tool used. Most papers investigating the determinants of body mass index (BMI) use either linear regression—to examine mean differences in BMI in different risk factor groups—or logistic regression—to examine if risk factor groups have higher odds of obesity. Neither of these options can straightforwardly determine whether risk factors are associated with differences across the distribution of the outcome in question (see Figure 1). Such differences may be important to better understand aetiology and inform policy. For example, since the population BMI distribution has become increasingly right-skewed from the 1980s (and its variance increased),^3^^,^^4^ risk factors that have contributed to this may have had a disproportionately stronger effect at the upper end of the BMI distribution (Figure 1B) (and/or simply increased in prevalence). In Rose’s seminal paper (see page 431),^5^ the ‘Population Strategy' was described as shifting the distribution of risk equivalently in the entire population (Figure 1A), with total health benefits potentially greater than targeting specific individuals of high risk (the ‘High Risk’ strategy). It is possible however that intervening on some risk factors which are applicable to the entire population may both shift the distribution of risk and reduce its skew.

As noted by authors recently in the epidemiological literature,^6^ quantile regression is an analytical tool that enables investigation of risk factor–outcome associations across the outcome distribution (i.e. beyond standard cut-points). The statistical underpinning has been described previously elsewhere, as have applied examples of its interpretation, and (beyond the scope of the current paper) technical work on heterogeneous treatment effects.^7–10^ Briefly, whereas linear regression estimates mean differences in outcomes across risk factor groups (which are likely identical in Figure 1A and B), and logistic regression compares odds of being above a threshold (odds ratios are both >1 in Figure 1A and B), quantile regression estimates the difference in a given quantile of the outcome distribution. For example, when comparing Figure 1B with Figure 1A, the median (50th quantile) differences are likely to be similar, yet differences in the 90th BMI quantile are notably higher Figure 1B.

Using quantile regression, we recently observed that absolute socio-economic inequalities in children’s BMI were substantially larger in higher BMI quantiles;^11^ mean BMI differences in the lowest vs highest socio-economic position (SEP) (in the cohort born in 2001 at 11 years) was 1.3 kg/m^2^ [95% confidence interval (CI): 0.9, 1.6]; the median difference was 0.98 kg/m^2^ (95% CI: 0.63, 1.33), yet the difference at the 90th quantile was 2.54 (1.85, 3.22). Similar findings have been observed in other studies in the UK, Spain, Norway and the USA (among men).^12–15^ Across the literature, there appears to be evidence for a phenomenon that does not seem to have been explicitly noted nor explained—in the minority of cases where the outcome distribution is explicitly investigated, associations between a myriad of risk factors and BMI are progressively larger at the upper ends of the BMI distribution. This includes genetic factors,^16^^,^^17^ behavioural factors (physical activity, sedentary behaviour and diet^18^^,^^19^), and family factors (maternal BMI or exercise).^18^^,^^20^ In this paper, we provide an illustrative example of this, examining multiple exposures in a single dataset, provide potential explanations and discuss potential implications for epidemiological research and policy.

## Demonstration

Data are from the 1958 British birth cohort study, a longitudinal study described in detail elsewhere,^21^ with prospective risk factors data and BMI measured at 45 years. Exposures were as follows: paternal social class at birth (categorized as manual vs non-manual given evidence for non-linearity); maternal weight at birth (<9 stone and ≥9 stone given evidence for non-linearity; 1 stone = 6.35 kg); general cognitive test score at 11 years (40 verbal and 40 non-verbal items converted to a *z*-score); and physical inactivity at 42 years [reported leisure activity at least once a month for most of the year (active) or less (inactive)]. Associations between these exposures and BMI were examined using linear regression and then conditional quantile regression, at the 10th, 25th, 50th (median), 75th and 90th quantiles. Models were mutually adjusted for each exposure, yet similar findings were found when conducting unadjusted analyses (data available upon request). We additionally adjusted for adult height to examine if this confounded our findings. Raw untransformed (kg/m^2^) BMI values were modelled to estimate absolute differences in BMI; additional models were conducted using log-transformed BMI to estimate relative (%) differences in BMI per increase in exposure. STATA 15 was used (StataCorp, 2017). Analytical syntax to reproduce these findings is available here: https://github.com/dbann/distributions_1958.

Table 1 shows associations between multiple established risk factors for high BMI: low childhood SEP (birth), high maternal weight (birth), low childhood general cognition (11 years) and adult physical inactivity (42 years). For each risk factor, the magnitude of associations was substantially larger at higher BMI quantiles. Associations of low SEP and low cognition with higher BMI were around three times larger at the 90th compared with the 10th quantile; effect estimates for maternal weight were ∼59% larger, whereas effect estimates for physical inactivity were null at lower quantiles and only found at higher BMI quantiles. The following section attempts to explain why such findings may exist and potential sources of bias, where possible using data from the 1958 cohort to investigate the plausibility of each explanation.

**Table 1.** Associations between risk factors for body mass index (kg/m^2^) at 45 years using both linear regression and quantile regression in the 1958 British birth cohort study^a^; standard errors in parentheses

Variables	Linear regression estimates (mean difference in BMI)	Quantile regression estimates (difference in BMI at below quantiles)				
		q10	q25	q50	q75	q90
Paternal social class (manual vs non-manual), birth	1.02***	0.63***	0.54***	0.80***	1.14***	1.90***
	(0.13)	(0.12)	(0.20)	(0.12)	(0.20)	(0.33)
Maternal weight (9 stone or more vs less), birth	1.16***	0.91***	1.01***	1.10***	1.35***	1.45***
	(0.12)	(0.16)	(0.16)	(0.14)	(0.23)	(0.30)
General cognition (per 1 lower SDS^b^), 11 years	0.43***	0.20***	0.36***	0.46***	0.57***	0.66***
	(0.064)	(0.064)	(0.073)	(0.083)	(0.12)	(0.14)
Physical exercise (inactive vs active), 42 years	0.66***	−0.24	0.083	0.58***	1.13***	1.64***
	(0.13)	(0.17)	(0.17)	(0.17)	(0.23)	(0.42)
Observations	6943	6943	6943	6943	6943	6943

*** *P* < 0.01.

a Models are mutually adjusted.

b SDS, standard deviation score.

## Why could risk factor–outcome associations be stronger at the upper end of the distribution?

### Heterogeneous (non-constant) causal effects of a single risk factor

Risk factors may have multiple contrasting causal effects on the outcome which differ across the outcome distribution. For example, low SEP has been shown to be associated with increased risk of both obesity (prevalence ∼20%) and risk of thinness (prevalence ∼ 6%)^22^ such that quantile regression estimates might show a negative relationship with BMI at the lower part of the distribution but a positive relationship with BMI at the upper part of the distribution. Indeed, in large datasets with sufficient numbers of thin participants, quantile regression estimates show reversal of the SEP–BMI association in the lower and upper end of the BMI distribution.^14^ Similarly, physical activity might reduce fat mass but increase muscle mass,^23^^,^^24^ such that physical activity might be related to lower body weight at the upper part of the distribution but related to higher body weight at the lower part of the distribution (due to the primary aim or effect of exercise being muscle gain/preservation rather than fat loss). A range of environmentally attributable risk factors may have stronger causal effects at the upper end of the BMI distribution—a recent twin study^25^ suggested that environmental effects on BMI may be stronger at the upper end, yet estimates of genetic effect stronger at the centre of the distribution.

Additionally, unmeasured risk factors may modify the effect of the risk factor of interest and lead to larger effects at the higher end of the distribution. Indeed, risk factors for obesity tend to cluster and do not act in isolation.^26^ As an example, individuals with higher BMI values are more likely than individuals with lower BMI values to have genetic variants that cause excessive weight gain. As suggested by the gene-by-environment literature, this genetic risk may result in the effect of poor diet, for example, being greater among individuals with higher BMI who have higher genetic risk for obesity. Environmental or behavioural factors could also work in the same way. For example, individuals with higher BMI values may live in areas with poorer dietary options such that the effects of socio-economic disadvantage are more pronounced at the upper end of the distribution. Some findings appear to support this suggestion—for instance, in the UK Biobank, estimated effects of genes on BMI were larger in more deprived areas.^27^

### Risk factor mismeasurement or confounding

Different effects sizes across the outcome distribution could be explained by the risk factor not measuring the construct of interest equivalently across the outcome distribution, or being confounded by other factors. For example, it is theoretically possible that individuals from low childhood social class backgrounds who have low BMI (rather than the anticipated high BMI), may in fact be a selected subset of participants who in fact are of higher SEP by some other measure (such as higher maternal education and/or family income). Thus, the extent of risk factor confounding may differ across the outcome distribution. This would lead to spuriously weaker associations at lower quantiles matching those observed in Table 1, driven by low correlations between the SEP indicator used and the construct of interest. We recommend that researchers test this possibility, for example by examining the convergent validity of the exposure across the outcome distribution. In our data, we did so by examining associations between father’s social class at birth and maternal education across BMI quintiles—reassuringly, correlations were found across the BMI distribution and were in fact stronger at lower quantiles [Spearman’s R (from lowest to highest BMI quintiles = 0.41, 0.39, 0.32, 0.35, 0.25)]. Confounding by adult height is also a possibility—BMI is constructed to create an index of weight that is uncorrelated with height, yet this may not function similarly across the BMI distribution. If BMI is associated with height at higher BMI values (and null at lower values), factors associated with both BMI and height could then appear to have stronger associations at upper BMI values. We found that height is negatively associated with BMI at upper quantiles, but that additional adjustment for height did not substantially affect the pattern of results for other exposures investigated (Supplementary Table S1, available as Supplementary data at *IJE* online).

### Absolute and relative differences

Findings could be an artefact attributable to the scale of the outcome measure used. Although it is possible that a given change in risk factor has a uniform effect across the BMI distribution—for example, a given diet intervention could lead to an equivalent 5 kg/m^2^ loss for everyone exposed (i.e. both those with average and high BMI values)—it may instead lead to a given percentage change (e.g. 5%). When examined on the absolute (kg/m^2^) scale, a diet that uniformly affects percent change in weight would seem to have a larger effect at the upper end of the distribution, yet an identical effect when examined on the relative scale (5% of BMI = 20 = 1; 5% of BMI = 30 = 1.5). Thus, it seems useful to examine the risk factor and BMI association at the upper end of the distribution on both absolute and relative scales. However, we demonstrate in Supplementary Table S2, available as Supplementary data at *IJE* online, that associations with the risk factors used and BMI are similar when BMI is modelled in relative (logged, %) terms.

### Outcome mismeasurement

Differential measurement error could theoretically induce stronger associations at the upper end of the distribution. For example, if the BMI and fat mass associations are stronger at higher BMI values (perhaps reflecting greater variance in fat rather than muscle mass in the population), and the exposure investigated is associated with fat but not lean mass, associations between the risk factor and BMI would be stronger at higher BMI values. However, studies examining associations between BMI and direct measures of fat mass find that the relationship is largely positive and linear, or in fact weaker at upper BMI quantiles.^28^^,^^29^ As such, this explanation is unlikely. Similar findings in our study were found with waist circumference as an outcome, providing evidence that findings are not an artifact caused by the indirect adiposity measure used (Supplementary Table S3, available as Supplementary data at *IJE* online).

## Implications and conclusions

Building on recent calls that researchers investigating descriptive trends in health examine both measures of average and distribution,^30^ we recommend that, in order to better understand the determinants of the distribution of population health, tools such as quantile regression could be used more frequently in aetiological epidemiology. This applies across many outcomes since population health (physical and mental) is ultimately thought to exist on a continuum, even when the measured constructs are quantified in binary or ordinal form.^2^ This is particularly so in obesity research, given the evidence for larger effect sizes at the upper parts of the BMI distribution and the limitations of conventional reliance on obesity cut-points, which leads to a loss of information and reduced statistical power. The uncertainty in the specific cut-points to use—particularly for direct measures of fat mass and childhood BMI measures—is further motivation for its use.

Solely estimating average effects (e.g. via linear regression) may lead to underestimation of the magnitude of effects in particular at-risk fractions of the population of interest—those in worse health. Investigation of this phenomenon in other outcomes requires sufficiently varied populations, and there are multiple methodological challenges to overcome in order to ensure that this is achieved. For example, those in worse health are typically most frequently lost to follow-up in longitudinal studies,^31^ and there have been declines in response rates to health surveys in recent decades.^32^ Additionally, biomedical outcomes may have detection limits at upper values, or be collected in sparse bins which impedes the precise estimation of effects at particular quantiles. As with conventional linear regression, causal interpretation of quantile regression estimates requires a series of strong assumptions (e.g. no unmeasured confounding). Less understood however, are how confounding and other sources of bias might act to lead to a particular pattern of results from quantile regression—such as increasingly large effect sizes at the upper outcome values.

How are understanding ‘distributional’ effects relevant for policy? If a risk factor has a causal effect on a health outcome, and its effect is heterogeneous—with increasingly larger effects at higher values (where health is worse)—then intervening on this risk factor may have greater health benefits than anticipated than when only examined in average terms. Thus, this information may be useful to inform evidence-based policy decision-making, including on which interventions should be scaled-up to promote health. Indeed, it has recently been suggested that clinical trials should report distributional changes in treatment groups in addition to reporting average differences.^33^ Our findings can be interpreted as being consistent with a population strategy for public health, and suggest that intervening on some risk factors that are applicable to an entire population may both shift the entire distribution of risk and reduce its skew. Alternatively, it is possible that some risk factors may lead to lower average BMI due to differences in the lower part of the BMI distribution; given suggestions that BMI has a J-shaped relationship with mortality,^34^ such factors may have worse (or net negative) effects on population health than anticipated when considering average BMI values alone. Methods such as quantile regression may therefore be particularly suitable where the outcome of interest has a non-linear effect on other health outcomes.^35^

## Supplementary Data

Supplementary data are available at *IJE* online.

## Funding

D.B. is supported by the Economic and Social Research Council (grant number ES/M001660/1) and the Academy of Medical Sciences/the Wellcome Trust ‘Springboard Health of the public in 2040’ Award [HOP001\1025]. W.J. is supported by a UK Medical Research Council (MRC) New Investigator Research Grant (MR/P023347/1), and acknowledges support from the National Institute for Health Research (NIHR) Leicester Biomedical Research Centre, which is a partnership between University Hospitals of Leicester NHS Trust, Loughborough University and the University of Leicester. E.F. is support by the Economic and Social Research Council (grant number ES/M001660/1).

## Supplementary Material

dyz245_Supplementary_Data

## References

[1] Porta M. A Dictionary of Epidemiology. Oxford: Oxford University Press, 2008.

[2] Keyes KM, Galea S. Population Health Science. Oxford: Oxford University Press, 2016.

[3] Flegal KM, Troiano RP. Changes in the distribution of body mass index of adults and children in the US population. Int J Obes 2000;24:807. 10.1038/sj.ijo.0801232 10918526

[4] Johnson W, Li L, Kuh D et al How has the age-related process of overweight or obesity development changed over time? Co-ordinated analyses of individual participant data from five United Kingdom birth cohorts. PLoS Med 2015;12:e1001828. 25993005 10.1371/journal.pmed.1001828 PMC4437909

[5] Rose G. Sick individuals and sick populations. Int J Epidemiol 2001;30:427–32. 11416056 10.1093/ije/30.3.427

[6] Beyerlein A. Quantile regression—opportunities and challenges from a user's perspective. Am J Epidemiol 2014;180:330–31. 24989240 10.1093/aje/kwu178

[7] Koenker R. Quantile Regression. Cambridge: Cambridge University Press, 2005.

[8] Angrist JD, Pischke J-S. Mostly Harmless Econometrics: An Empiricist's Companion. Princeton: Princeton University Press, 2008.

[9] Athey S, Imbens G. Recursive partitioning for heterogeneous causal effects. Proc Natl Acad Sci USA 2016;113:7353–360. 27382149 10.1073/pnas.1510489113 PMC4941430

[10] Koenker R, Bassett G Jr Regression quantiles. Econometrica 1978;46:33–50.

[11] Bann D, Johnson W, Li L et al Socioeconomic inequalities in childhood and adolescent body-mass index, weight, and height from 1953 to 2015: an analysis of four longitudinal, observational, British birth cohort studies. Lancet Public Health 2018;3:e194. 29571937 10.1016/S2468-2667(18)30045-8 PMC5887082

[12] Gebremariam MK, Arah OA, Lien N et al Change in BMI Distribution over a 24‐year period and associated socioeconomic gradients: a quantile regression analysis. Obesity 2018;26:769–75. 29498224 10.1002/oby.22133

[13] Rodriguez-Caro A, Vallejo-Torres L, Lopez-Valcarcel B. Unconditional quantile regressions to determine the social gradient of obesity in Spain 1993–2014. Int J Equity Health 2016;15:175. 27756299 10.1186/s12939-016-0454-1 PMC5070139

[14] White J, Rehkopf D, Mortensen LH. Trends in Socioeconomic inequalities in body mass index, underweight and obesity among English children, 2007? 2008 to 2011? 2012. PLoS One 2016;11:e0147614. 26812152 10.1371/journal.pone.0147614 PMC4727904

[15] Liu SY, Kawachi I, Glymour MM. Education and inequalities in risk scores for coronary heart disease and body mass index: evidence for a population strategy. Epidemiology 2012;23:657–64. 22814529 10.1097/EDE.0b013e318261c7cc

[16] Mitchell JA, Hakonarson H, Rebbeck TR et al Obesity‐susceptibility loci and the tails of the pediatric BMI distribution. Obesity 2013;21:1256–260. 23408508 10.1002/oby.20319 PMC3661695

[17] Beyerlein A, von Kries R, Ness AR et al Genetic markers of obesity risk: stronger associations with body composition in overweight compared to normal-weight children. PLoS One 2011;6:e19057. 21526213 10.1371/journal.pone.0019057 PMC3078148

[18] Beyerlein A, Toschke AM, von Kries R. Risk factors for childhood overweight: shift of the mean body mass index and shift of the upper percentiles: results from a cross-sectional study. Int J Obes 2010;34:642. 10.1038/ijo.2009.301 20084072

[19] Azagba S, Sharaf MF. Fruit and vegetable consumption and body mass index: a quantile regression approach. J Prim Care Community Health 2012;3:210–20. 23803782 10.1177/2150131911434206

[20] Dahly DL, Li X, Smith HA et al Associations between maternal lifestyle factors and neonatal body composition in the Screening for Pregnancy Endpoints (Cork) cohort study. Int J Epidemiol 2018;47:131–45. 29136159 10.1093/ije/dyx221

[21] Power C, Elliott J. Cohort profile: 1958 British birth cohort (National Child Development Study). Int J Epidemiol 2006;35:34–41. 16155052 10.1093/ije/dyi183

[22] Pearce A, Rougeaux E, Law C. Disadvantaged children at greater relative risk of thinness (as well as obesity): a secondary data analysis of the England National Child Measurement Programme and the UK Millennium Cohort Study. Int J Equity Health 2015;14:61. 26242408 10.1186/s12939-015-0187-6 PMC4524014

[23] Bann D, Kuh D, Wills AK et al Physical activity across adulthood in relation to fat and lean body mass in early old age: findings from the Medical Research Council National Survey of Health and Development, 1946-2010. Am J Epidemiol 2014;179:1197. 24722997 10.1093/aje/kwu033 PMC4010186

[24] Peterson MD, Sen A, Gordon PM. Influence of resistance exercise on lean body mass in aging adults: a meta-analysis. Med Sci Sports Exerc 2011;43:249–58. 20543750 10.1249/MSS.0b013e3181eb6265 PMC2995836

[25] Tsang S, Duncan GE, Dinescu D et al Differential models of twin correlations in skew for body-mass index (BMI). PLoS One 2018;13:e0194968. 29590176 10.1371/journal.pone.0194968 PMC5874062

[26] Noble N, Paul C, Turon H et al Which modifiable health risk behaviours are related? A systematic review of the clustering of Smoking, Nutrition, Alcohol and Physical activity (‘SNAP’) health risk factors. Prev Med 2015;81:16–41. 26190368 10.1016/j.ypmed.2015.07.003

[27] Tyrrell J, Wood AR, Ames RM et al Gene–obesogenic environment interactions in the UK Biobank study. Int J Epidemiol 2017;46:559–75. 28073954 10.1093/ije/dyw337 PMC5837271

[28] Kyle UG, Schutz Y, Dupertuis YM et al Body composition interpretation: contributions of the fat-free mass index and the body fat mass index. Nutrition 2003;19:597–604. 12831945 10.1016/s0899-9007(03)00061-3

[29] Gallagher D, Heymsfield SB, Heo M et al Healthy percentage body fat ranges: an approach for developing guidelines based on body mass index. Am J Clin Nutr 2000;72:694–701. 10966886 10.1093/ajcn/72.3.694

[30] Razak F, Subramanian S, Sarma S et al Association between population mean and distribution of deviance in demographic surveys from 65 countries: cross sectional study. BMJ 2018;362:k3147. 30076132 10.1136/bmj.k3147 PMC6073428

[31] Stafford M, Black S, Shah I et al Using a birth cohort to study ageing: representativeness and response rates in the National Survey of Health and Development. Eur J Ageing 2013;10:145–57. 23637643 10.1007/s10433-013-0258-8 PMC3637651

[32] Mindell J, Giampaoli S, Goesswald A et al Sample selection, recruitment and participation rates in health examination surveys in Europe - experience from seven national surveys. BMC Med Res Methodol 2015;15:78. 26438235 10.1186/s12874-015-0072-4 PMC4595185

[33] Subramanian S, Kim R, Christakis NA. The “average” treatment effect: A construct ripe for retirement. A commentary on Deaton and Cartwright. Soc Sci Med 2018;210:77. 29724462 10.1016/j.socscimed.2018.04.027

[34] Bhaskaran K, dos-Santos-Silva I, Leon DA et al Association of BMI with overall and cause-specific mortality: a population-based cohort study of 3· 6 million adults in the UK. Lancet Diabetes Endocrinol 2018;6:944–53. 30389323 10.1016/S2213-8587(18)30288-2 PMC6249991

[35] Rehkopf DH Commentary: quantile regression for hypothesis testing and hypothesis screening at the dawn of big data. Epidemiology 2012;23:665–67. 22872112 10.1097/EDE.0b013e318261f7be

