# Supplementary material for: Determinants of the population health distribution: an illustration examining body mass index
Source: Int J Epidemiol. 2020 Jan 13;49(3):731–7. doi: 10.1093/ije/dyz245 (PMC7394943; doi:10.1093/ije/dyz245)
Supplement: dyz245_Supplementary_Data [file dyz245_supplementary_data.docx]

**Supplementary Tables**

Table S1. Associations between risk factors for body mass index at 44/45y using both linear regression and quantile regression in the 1958 British birth cohort study, adjusted for adult height

|  | Linear regression estimates (mean difference in BMI) | Quantile regression estimates  (difference in BMI at below quantiles) | | | | |
| --- | --- | --- | --- | --- | --- | --- |
| VARIABLES |  | q10 | q25 | q50 | q75 | q90 |
|  |  |  |  |  |  |  |
| Paternal social class (manual vs non-manual), birth | 1.02*** | 0.71*** | 0.62*** | 0.77*** | 1.17*** | 1.94*** |
|  | (0.13) | (0.13) | (0.13) | (0.12) | (0.19) | (0.33) |
| Maternal weight (9 stone or more vs less), birth | 1.16*** | 0.84*** | 0.78*** | 1.01*** | 1.42*** | 1.62*** |
|  | (0.12) | (0.15) | (0.12) | (0.099) | (0.19) | (0.28) |
| General cognition (per 1 lower SDS), 11y | 0.43*** | 0.19*** | 0.36*** | 0.46*** | 0.53*** | 0.62*** |
|  | (0.064) | (0.073) | (0.067) | (0.073) | (0.079) | (0.14) |
| Physical exercise (inactive vs active), 42y | 0.67*** | -0.15 | 0.15 | 0.59*** | 1.14*** | 1.35*** |
|  | (0.13) | (0.16) | (0.13) | (0.18) | (0.22) | (0.26) |
| Height at 45y (per 1m higher) | 0.13 | 5.31*** | 5.67*** | 3.54*** | -2.77*** | -8.21*** |
|  | (0.64) | (0.73) | (0.39) | (0.68) | (0.89) | (1.24) |
|  |  |  |  |  |  |  |
| Observations | 6,943 | 6,943 | 6,943 | 6,943 | 6,943 | 6,943 |

Standard errors in parentheses

*** p<0.01, ** p<0.05, * p<0.1

Note: models are mutually adjusted. BMI = body mass index. SDS = standard deviation score.

Table S2. Associations between risk factors for body mass index at 45y (logged) using both linear regression and quantile regression in the 1958 British birth cohort study

|  | Linear regression estimates (mean difference in BMI) | Quantile regression estimates  (difference in BMI at below quantiles) | | | | |
| --- | --- | --- | --- | --- | --- | --- |
| VARIABLES |  | q10 | q25 | q50 | q75 | q90 |
|  |  |  |  |  |  |  |
| Paternal social class (manual vs non-manual), birth | 0.035*** | 0.029*** | 0.024*** | 0.031*** | 0.040*** | 0.058*** |
|  | (0.0046) | (0.0054) | (0.0060) | (0.0044) | (0.0079) | (0.0096) |
| Maternal weight (9 stone or more vs less), birth | 0.042*** | 0.042*** | 0.042*** | 0.042*** | 0.046*** | 0.043*** |
|  | (0.0041) | (0.0069) | (0.0066) | (0.0042) | (0.0068) | (0.0079) |
| General cognition (per 1 lower SDS), 11y | 0.015*** | 0.0091*** | 0.015*** | 0.017*** | 0.019*** | 0.020*** |
|  | (0.0022) | (0.0032) | (0.0030) | (0.0023) | (0.0025) | (0.0050) |
| Physical exercise (inactive vs active), 42y | 0.021*** | -0.011 | 0.0032 | 0.022*** | 0.038*** | 0.048*** |
|  | (0.0047) | (0.0085) | (0.0056) | (0.0046) | (0.0077) | (0.0098) |
|  |  |  |  |  |  |  |
| Observations | 6,943 | 6,943 | 6,943 | 6,943 | 6,943 | 6,943 |

Standard errors in parentheses

*** p<0.01, ** p<0.05, * p<0.1

Note: models are mutually adjusted. BMI = body mass index. SDS = standard deviation score.

Table S3. Associations between risk factors for waist circumference (cm) at 45y using both linear regression and quantile regression in the 1958 British birth cohort study

|  | Linear regression estimates (mean difference in waist circumference) | Quantile regression estimates  (difference in waist circumference at below quantiles) | | | | |
| --- | --- | --- | --- | --- | --- | --- |
| VARIABLES |  | q10 | q25 | q50 | q75 | q90 |
|  |  |  |  |  |  |  |
| Paternal social class (manual vs non-manual), birth | 1.65*** | 0.78 | 1.64*** | 1.19*** | 1.64*** | 3.16*** |
|  | (0.36) | (0.59) | (0.55) | (0.40) | (0.53) | (0.66) |
| Maternal weight (9 stone or more vs less), birth | 3.52*** | 2.58*** | 3.32*** | 3.85*** | 3.81*** | 3.94*** |
|  | (0.32) | (0.55) | (0.63) | (0.55) | (0.46) | (0.51) |
| General cognition (per 1 lower SDS), 11y | 1.69*** | 1.21*** | 1.78*** | 1.65*** | 1.75*** | 2.23*** |
|  | (0.18) | (0.20) | (0.16) | (0.18) | (0.20) | (0.24) |
| Physical exercise (inactive vs active), 42y | 1.62*** | 0.38 | 0.79 | 1.19** | 2.37*** | 2.24*** |
|  | (0.37) | (0.56) | (0.54) | (0.49) | (0.45) | (0.60) |
|  |  |  |  |  |  |  |
| Observations | 7,016 | 7,016 | 7,016 | 7,016 | 7,016 | 7,016 |

Standard errors in parentheses

*** p<0.01, ** p<0.05, * p<0.1

Note: models are mutually adjusted. SDS = standard deviation score.
